# Human Endometrial Microbiota at Term of Normal Pregnancies

**DOI:** 10.3390/genes10120971

**Published:** 2019-11-26

**Authors:** Claudia Leoni, Oronzo Ceci, Caterina Manzari, Bruno Fosso, Mariateresa Volpicella, Alessandra Ferrari, Paola Fiorella, Graziano Pesole, Ettore Cicinelli, Luigi Ruggiero Ceci

**Affiliations:** 1 Department of Biosciences, Biotechnologies and Biopharmaceutics, University of Bari “A. Moro”, Via Orabona 4, 70126 Bari, Italy; c.leoni@ibiom.cnr.it (C.L.); mariateresa.volpicella@uniba.it (M.V.); g.pesole@ibiom.cnr.it (G.P.); 2 2nd Unit of Obstetrics and Gynecology, Department of Biomedical Science and Human Oncology, University of Bari “A. Moro”, Piazza G. Cesare, 70124 Bari, Italy; oronzoruggiero.ceci@uniba.it (O.C.); alessandraferrari.gineco@gmail.com (A.F.); fiorella.paola88@gmail.com (P.F.); 3 Institute of Biomembranes, Bioenergetics and Molecular Biotechnologies, CNR, Via Amendola 122/O, 70126 Bari, Italy; c.manzari@ibiom.cnr.it (C.M.); b.fosso@ibiom.cnr.it (B.F.)

**Keywords:** endometrium, microbiota, metabarcoding, metagenomics

## Abstract

The endometrium is a challenging site for metagenomic analysis due to difficulties in obtaining uncontaminated samples and the limited abundance of the bacterial population. Indeed, solid correlations between endometrial physio-pathologic conditions and bacteria compositions have not yet been firmly established. Nevertheless, the study of the endometrial microbiota is of great interest due to the close correlations between microbiota profiles, women’s health, and successful pregnancies. In this study, we decided to tackle the study of the endometrial microbiota through analysis of bacterial population in women subjected to elective caesarean delivery. As a pilot study, a cohort of 19 Caucasian women at full term of normal pregnancy and with a prospection of elective caesarean delivery was enrolled for endometrium sampling at the time of caesarean section. Sampling was carried out by endometrial biopsy soon after the delivery of the newborn and the discharge of the placenta and fetal membranes from the uterus. Bacterial composition was established by a deep metabarcoding next generation sequencing (NGS) procedure addressing the V5–V6 hypervariable region of the 16S rRNA gene. Amplicon sequences were analysed by bioinformatic procedures for denoising and taxonomic classification. The RDP database was used as 16S rRNA reference collection. Metabarcoding analysis showed the presence of a common bacterial composition, including six genera classifiable within the human microbiota (*Cutibacterium*, *Escherichia*, *Staphylococcus*, *Acinetobacter*, *Streptococcus*, *Corynebacterium*), that could be part of the core endometrial microbiota under the specific conditions examined. These results can provide useful information for future studies on the correlations between bacteria and successful pregnancies.

## 1. Introduction

The human microbiota consists of various microbial populations distributed in the different sites of the body. By overcoming the limited possibility of culturing most of the microbes in the laboratory, advanced next generation sequencing (NGS) technologies today allow an almost complete identification of specific microbiota compositions either by whole microbiome shotgun sequencing or by DNA metabarcoding analysis [1]. Metagenomic studies pointed out that the human body hosts a large number of microbial cells, at least equal to the human counterpart and corresponding to a much higher number of genes and strongly influencing our health and disease conditions [2]. It has been ascertained that numerous diseases (such as inflammatory bowel disease, cancer, and major depressive disorder) are correlated with microbiota composition, its functional activity, and its interactions with human immune, endocrine, and nervous systems [3,4].

The first large-scale metagenomic investigations on human microbiota focused on body sites directly exposed to external sources of colonization (skin, mouth, vagina, gut, etc.) in the course of the Human Microbiome Project (HMP) (The Human Microbiome Project Consortium, 2012). Nevertheless, even sites not directly exposed to external environments can harbour specific microbiotas that in turn influence the host physio-pathologic conditions. The uterus is one of these sites. Nowadays, evidence from NGS investigations strongly supports the existence of a uterine microbiota and metagenomic approaches are gaining momentum in the analyses of the human endometrial microbiota under different conditions. Several reviews and commentary articles are already available on this specific topic [5,6,7,8,9,10,11,12]. Besides advances in the knowledge of the microbiota composition and its origin, these studies can also help to establish possible correlations between microbiota composition and uterus specific physiologic or pathologic conditions, including pregnancy, sterility, and conditions in which assisted reproductive technologies (ART) are adopted.

Uterine microbiomes were investigated by NGS approaches in women subjected to ART [13,14,15], with either recurrent reproductive failure [16], repeated implantation failure [17], or affected by endometriosis [18] and endometrial polyps (with and without and chronic endometritis) [19]. Most of these studies were carried out by using sampling procedures that adopted trans-cervical sampling devices. A more limited number of studies, in cases of women subjected to surgical uterus removal, adopted direct in-utero sampling [20,21,22,23,24]. 

All these studies did not define a clear scenario of the human uterine microbiota and its dynamics in either physiological or pathological conditions, and some discrepancies can also be observed among the various analyses (for critical evaluations see the above cited reviews). It must be underlined, however, that the characterization of the uterine microbiome is particularly difficult, mainly due to possible contaminations occurring during trans-vaginal sampling and the low bacterial abundance of the site [9,10,12,24]. Clearly, further studies are required to establish the microbiota composition(s) of this site in its various physiological and pathological conditions. 

In particular, it would be interesting to study the uterine microbial composition during pregnancy and evaluate any possible correlation with its evolution. In this pilot study, we focused on the characterization of the uterine microbiomes of women subjected to elective caesarean deliveries at full term of normal pregnancies, since this condition allows direct sampling of the endometrial tissue while avoiding vaginal contaminations. Results highlight the consistent presence of a limited number of bacterial genera, which in part confirm previous studies on human endometrial microbiota. In particular, the presence of *Cutibacterium* (formerly *Propionibacterium* [25]) and *Lactobacillus* genera is discussed.

## 2. Materials and Methods

### 2.1. Study Cohort and Sample Collection

Of the caesarean deliveries that occurred at the 2nd Unit of Obstetrics and Gynecology, University of Bari, Polyclinic University-Hospital, Bari (Italy), 19 cases were selected for this study. All the deliveries were elective caesarean sections at full term of physiological single pregnancies. Patients were Italian residents with Caucasian origin. Exclusion criteria were the inability to provide informed consent, less than 18 years of age, premature rupture of the membranes, use of antibiotic drugs during the pregnancy, and positive culture-based screening at 35–37 weeks of gestation on vaginal-rectal swab. 

Endometrial samples were collected under strict aseptic conditions in an operating room with a ceiling air filtration system to ensure the reduction of contaminations from airborne microorganisms. Sampling was carried out in triplicate by endometrial biopsy in the opposite wall with respect to placental insertion at about 10–15 cm from the internal cervical ostium soon after the discharge of placenta and fetal membranes. Biopsies were obtained by lifting the endometrium with a new sterile clamp and then cutting samples of about 5 × 5 mm with new sterile scalpels. Biopsies were immediately frozen and stored at −80 °C until DNA extraction. 

The study was approved by the Institutional Ethical Committee of the Polyclinic University Hospital, Bari (Italy) and informed consent was obtained from each patient. There were no conflicts of interest associated with this study.

### 2.2. DNA Extraction

Metagenomic DNA from endometrial samples was extracted by using the Fast DNA Spin Kit for Soil (MP Biomedicals, Irvine, CA, USA), according to the manufacturer’s instructions. The final product was 100 μL of application ready DNA. A negative control was carried out using the reagents and tubes supplied with the kit in a fake extraction procedure. The quality and concentration of the DNA extracts were determined by 1% agarose gel electrophoretic analysis and by spectrophotometric measurements at 260, 280, and 230 nm using the NanoDrop^®^ ND-1000 Spectrophotometer (ThermoFisher Scientific Inc., Milan, Italy). DNA samples were stored at −20 °C until further analyses.

### 2.3. Amplicon Library Preparation and Illumina-Based Sequencing

Bacteria identification was performed by sequencing analysis of amplicon libraries of the V5–V6 hypervariable regions of the 16S rRNA gene [25], using the primers B-V5 and A-V6 [26]: 

B-V5: 5′- TCGTCGGCAGCGTCAGATGTGTATAAGAGACAG/ATTAGATACCCYGGTAGTCC-3′; 

A-V6: 5′- GTCTCGTGGGCTCGGAGATGTGTATAAGAGACAG/ACGAGCTGACGACARCCATG-3′.

The first part of each primer, before the slash, corresponds to the Nextera transposase sequence required by the Illumina protocol. The 16S rRNA gene corresponding part of each primer is underlined. The strategy used to prepare the 16S rRNA amplicon-based library was described in detail in [27]. From each sample, 100 ng of DNA was extracted and used for a two-step amplification reaction to yield amplicons having at their termini an Illumina adapter (P7 or P5) and an Illumina Nextera index sequence, as required by the dual index sequencing approach. RNase/DNase-free Molecular Biology Grade water (Ambion) was used as a negative control of PCR amplification. Purified amplicons were pooled in an equimolar ratio and subjected to a 2 × 250 bp paired-end sequencing on the Illumina MiSeq platform. To increase the genetic diversity, as required by the MiSeq platform, a phage PhiX genomic DNA library was added to the mix and co-sequenced.

### 2.4. Bioinformatic Analysis

The obtained Illumina MiSeq reads were analyzed by using a bioinformatic procedure including the two main steps of denoising and taxonomical classification. The first one relies on the Amplicon Sequence Variants (ASVs) inference and the latter on the taxonomic annotation of the inferred ASVs. In particular, raw paired-end (PE) reads were treated with trim-galore! (https://www.bioinformatics.babraham.ac.uk/projects/trim_galore/) to remove Illumina adaptors. Following, the ASVs inference was performed by applying the DADA2 procedure [28]. The obtained ASVs were taxonomically annotated in BioMaS [29] by using release 11.5 of the RDP database [30] as the 16S rRNA reference collection and the NCBI taxonomy. In particular, the ASVs sequences were aligned to the reference collection by using Bowtie2 [31] and the resulting alignments were filtered according to query coverage (≥70%) and identity percentage (≥90%). The taxonomic classification was performed by using TANGO [32]. For ASV sequences obtaining matches with identity percentages equal or higher than 97%, the taxonomic classification at species level was accepted [33]; otherwise, they were classified to higher taxonomic ranks. Contaminants were identified and removed by using decontam [34]. Rarefaction curves were inferred by using the Vegan R Package [35] and used to define the rarefaction value used to normalize the ASVs table. Following, the Shannon and the Faith indexes (α diversity) were inferred by applying the R-package phyloseq [36] on the rarefied ASVs table. Prior to the β-diversity, the presence of a batch effect was evaluated by using the ComBat function of the SVA R package [37,38]. The Non-metric Multidimensional Scaling (NMDS) plots describing the diversity between the samples (i.e., β-diversity) based on the weighted and unweighted UniFrac and Bray–Curtis dissimilarity matrices were obtained by using phyloseq [36].

## 3. Results

### 3.1. Data Collection and Statistical Analysis

Sampling of endometrial biopsies was carried out between March and October 2018. Details on pregnancy conditions for each patient are reported in Table 1.

To analyze the bacterial population in each sample, we performed amplification and sequencing of the V5–V6 hypervariable regions of the 16S rRNA gene [25]. After the trimming of raw sequences and removal of chimera and Phix sequences, about 2.4 million of paired-end (PE) reads were obtained. 57 distinct sequencing raw data (corresponding to 19 patients in triplicate) were deposited in the SRA (Short Read Archive) repository with accession number PRJNA557586.

By taking into account the rarefaction curves (Figure S1) and the number of sequences for each sample (Table S1), 15,000 was taken as the rarefaction deep value to correctly profile all the investigated microbiomes. According to this threshold, 52 sequencing samples (instead of 57) were used in the subsequent analyses.

**Figure 1:**
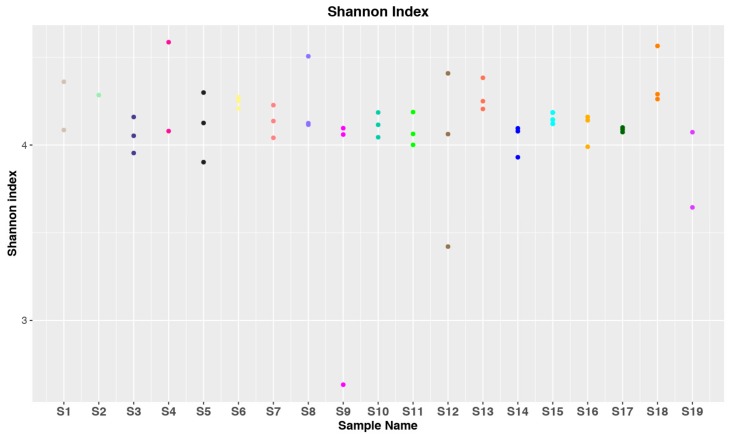
Shannon diversity index (H) values related to the bacterial 16S rRNA gene amplicon sequences for the endometrial samples. S1–S19 indicate the different subjects. Colored dots correspond to a single V5–V6 amplicon sequencing sample.

The Shannon diversity index (H) for the samples of the different patients is shown in Figure 1. The differences observed for the same subject may be a consequence of the different sampling regions within the uterine cavity. In any case, most of the samples (45/52) lay in a restricted range of H values (about 4–4.5) indicating a relative homogeneity of the samples both in bacterial diversity and their relative abundances. Only in one sample of the S9 subject was a very low H value observed, corresponding to a sample dominated by the *Pseudomonas* genus. The homogeneity of the bacterial diversity in the different samples is also illustrated by the Faith index (Figure S2) ranging within a limited range (about 10–20) for the 52 samples. The Faith index refers to the Phylogenetic diversity (PD), a measure of biodiversity based on a phylogenetic tree defined by Faith in 1992 [39]. Considering a set of species, or in the specific case of metabarcoding, a set of ASVs or operational taxonomic units (OTUs), the PD is equal to the sum of the branch length in the tree spanning the members of the set.

To analyze the β-diversity among samples the weighted and unweighted UniFrac and Bray–Curtis dissimilarity matrices were obtained by using phyloseq [36] and plotted as NMDS. Regardless of the used dissimilarity measures, it is possible to observe a tight clustering of almost all samples, confirming the relative homogeneity of the samples (Figure S3).

### 3.2. Taxonomic Distribution

**Figure 2:**
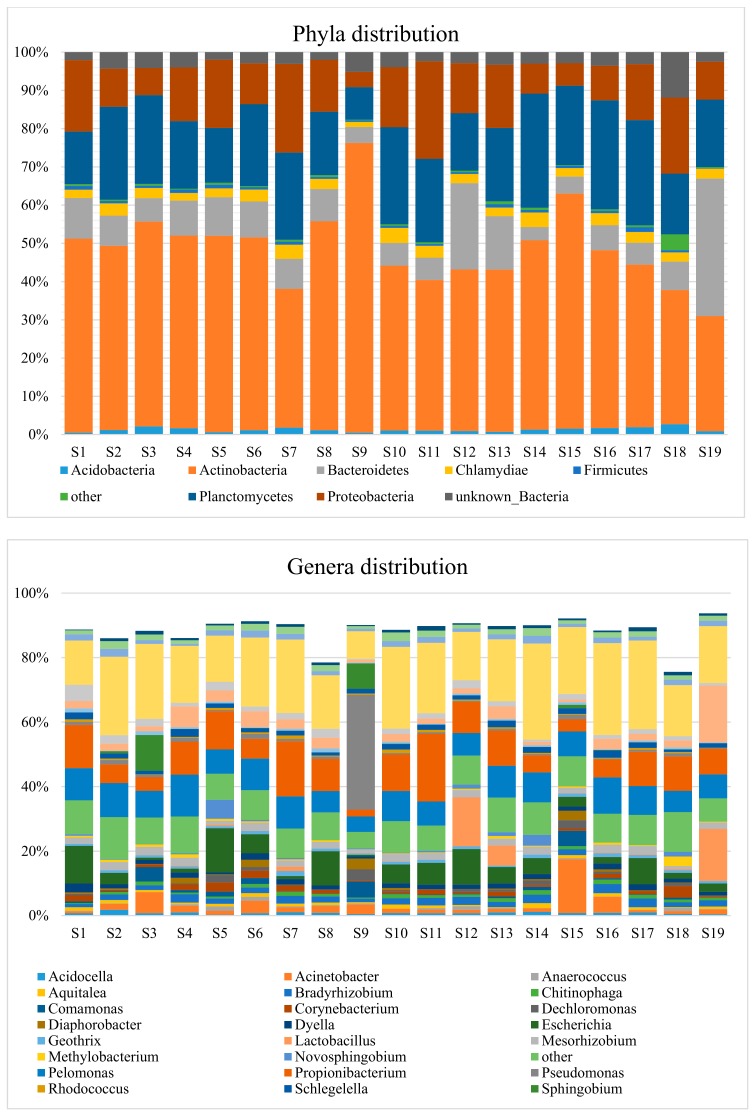
Bar charts of taxonomic classification of bacteria identified in endometrial samples at phylum and genus levels (obtained as mean values for the three replicates).

The taxonomic distributions of endometrial bacteria are given in Figure 2 for the phylum and genus ranks. Only groups with relative abundances ≥1.0% were reported (the relative genera abundances for each patient are given in Table S2). In the graph of genera distribution, a number of sequences that could not be assigned were reported at the highest identified taxonomic level. Unknown bacteria represents the most abundant group (20.22%) and unknown *Acidobacteriales*, unknown *Deltaproteobacteria*, and unknown *Rhizobiales* are present at lower levels, 1.54%, 1.71% and 0.80%, respectively. Of the 26 genera reported in Figure 2, 11 were present in more than 50% of the examined subjects (Table 2). Among them, the presence of contaminant bacteria is quite evident considering the detection of genera such as *Pelomonas*, *Mesorizhobium*, *Bradyrhizobium*, *Schlegelella,* and *Dyella* (Table 2). Their diffusion among the subjects is homogeneous and with limited variations with *Pelomonas* at 4.78%–12.8%, *Mesorizhobium* at 0.98%–2.64%, *Bradyrhizobium* at 0.95%–2.80%, *Schlegelella* at 0.76%–2.45%, and *Dyella* at 0.60%–2.76%. It is interesting to note that the genus *Lactobacillus* is present only in about one-fifth of the patients and with a high degree of variability (1.40%–16.18%).

## 4. Discussion

We have determined the bacterial composition of human endometrium in 19 women subjected to elective caesarean delivery by a deep metabarcoding NGS procedure addressing the V5–V6 hypervariable region of the 16S rRNA gene. All the patients enrolled in the study did not show any particular health condition requiring the use of antibiotics and reached full term pregnancy. The sampling procedure directly in-utero prevented possible contaminations from the vaginal microbiota, a major problem in many analysis of uterine microbiota [9,10,12,24].

DNA purification, preparation, and sequencing of amplicon libraries and sequences analysis were carried out according to consolidated procedures [27,40]. The number of obtained sequences (Table S1) was high enough to cover the bacterial diversity for each sample (Figure 1). Nevertheless, five samples with the number of sequences below the established threshold of 15,000 were discharged. Analysis of α-diversity, carried out by the Shannon method [41], provided H index values in a restricted range (about 4–4.5) for almost all the samples (Figure 1). It appears that the three distinct endometrial samples of each subject have a different bacterial community, albeit with limited variations. Also β-diversity analysis showed a limited dispersion for most of the sequence samples (Figure S2). The observation that the bacterial composition can change, even in close areas of the uterine cavity, must be considered for future comparative studies on the characterization of uterine microbiota.

The bacterial distribution at the genus level is of particular interest (Figure 2). Among the relatively low number of detected genera (26), 10 appear almost ubiquitous, being present in over 84% of the subjects (Table 2). These figures are not yet perfectly indicative of the composition of the human endometrial microbiome for at least two reasons. These are the high percentage of ASVs placed in the unassigned groups (about 24%) and the presence of contaminant bacteria. 

Regarding the unassigned sequences, it should be stressed that endometrium is a very under-investigated niche especially if compared with other sites, such as the gut. This is an issue because we do not possess adequate information in reference databases to fully taxonomically annotate the endometrial microbiome. In this regard, it may represent a potentially interesting field of investigation for the characterization of the genomes of unknown bacteria and their association with the healthy state of the female reproductive tract. 

Contamination from environmental bacteria in microbiota analysis of endometrial samples is a well-known problem that can be explained by taking into account the low abundance of bacteria in the human uterus [9,10,12,24]. In fact, even working under strict sterile conditions, contaminants are still evident. Sources of contamination can be solutions, reagents, instrumentation, and the molecular biology kits used in the analysis [9,10,12,24]. Furthermore, it must be considered that the incidence of contaminants becomes even more relevant when analysis is carried out with high sensitivity techniques such as NGS [42]. In our analyses, even when sampling was carried out by sterile procedures in an aseptic environment and DNA manipulations were conducted using standard sterile protocols (including negative controls), contamination from environmental bacteria was still detectable. Additional negative controls in the course of the sampling procedure could have clarified the origin of the bacterial contaminations. At the same time, contaminations from other tissues of the patients (e.g., peritoneum, myometrium) can be excluded as surgeons sampled directly from the decidua into the uterine cavity.

Interestingly enough, six genera classifiable within the human microbiota (*Cutibacterium*, *Escherichia*, *Staphylococcus*, *Acinetobacter*, *Streptococcus*, *Corynebacterium*) are shared by most of the samples (Table 2). They may be part of the human core endometrial microbiota, at least under the particular cases analyzed (pregnant women subjected to elective at full term cesarean delivery). These genera have also been reported in other studies on human endometrial microbiota but with different distributions, probably depending on the different physio-pathological conditions and experimental procedures [5,6,7,9,10]. 

The high abundance of the *Cutibacterium* (formerly *Propionibacterium*) genus detected in our analysis deserves particular attention. This genus was not described in most of the endometrial microbiota described so far. Nevertheless, it was also reported in a limited number of studies. In a metagenomic analysis of placenta from more than 300 women, *Propionibacterium acne* was found among the prevailing species [43]. In this study, the authors also found that placental microbiomes clustered with the hosts’ oral microbiomes and proposed a possible hematogenous origin. The *Propionibacterium* genus was also detected in the course of a study aimed at identifying bacteria transfer dynamics in newborns. It resulted that *Enterobacter*, *Escherichia*/*Shigella*, and *Propionibacterium* were, in order, the genera with the highest abundances, both in the placenta and in amniotic fluid [44]. Chen and collaborators [21] analyzed the microbiota composition in the lower and upper female reproductive tracts during proliferative and secretory phases and found that OTUs that led to optimal classification between the two phases also included *Propionibacterium acnes*, with increased abundance during the secretory phase. More recently, in a comparative study of the endometrial metagenomes of women with menorrhagia and dysmenorrhea, the *Propionibacterium* genus was identified with abundance levels even higher than 10% in subjects with dysmenorrhea and assuming exogenous progestins [45]. Paired analysis of endocervix samples from the same subjects did not show the presence of *Propionibacterium*. The presence of this genus therefore seems probably linked to particular woman conditions, including late pregnancy.

In the present study, the genus *Lactobacillus* was detected with levels of abundance between 0% and 16%. A high variability of *Lactobacillus* abundance can also be found in other studies on the characterization of the human endometrial microbiome, although it has not been adequately reported so far. In fact, only by considering studies performed with direct sampling in-utero (in order to exclude contamination from the vagina), we can find high variability of *Lactobacillus* in at least two other cases. Miles and collaborators [22] analyzed the endometrial microbiota in nine patients subjected to total hysterectomy and found the *Lactobacillus* genus varying in the 0%–100% range. In another study, the endometrial microbiota of 80 women subjected to laparoscopy was analyzed after swab sampling of the tissue [21]. Although the authors reported an average presence of the genus *Lactobacillus* at 30.6%, the data in the Supplementary Materials shows an irregular distribution, with a very wide variability among the different subjects ranging from 0% to >95%. Taken together, these data indicate that for the cases under investigation *Lactobacillus* can be present in the human endometrium, but without being a part of the core microbiota. In the 19 patients examined in our study the abundance of the *Lactobacillus* genus is below 1% in 15 subjects, strengthening the hypothesis of a uterine microbiota generally devoid of such genus (at least in the examined conditions). Nevertheless, the possibility that in some conditions *Lactobacilli* can ascend from the vagina to the uterus would explain the relatively high abundance values found in four cases (from 1.40% to 16.18%). Apparently, no particular health or clinical conditions differentiated these four patients from the other examined women. In conclusion, while an endometrium with high levels of *Lactobacilli* is required for successful reproductive function [15], our data indicate that during pregnancy, the presence in the decidua of a series of different bacteria, not always including *Lactobacilli*, is associated with normal pregnancies.

## Figures and Tables

**Table 1:** Pregnancy-related characteristics of enrolled subjects.

ID	Age	BMI	Weeks at Birth	Previous Births/Abortions	Indication for Cesarean Section
S1	35	27.34	39	1/0	Previous cesarean section
S2	49	20.76	40	0/2	Voluminous Cervical Leiomyoma
S3	33	23.88	40	0/0	Previous laparoscopic myomectomy
S4	22	21.48	39	1/0	Previous cesarean section
S5	39	24.97	39	1/1	Previous cesarean section
S6	25	31.49	41	0/0	Hereditary angioedema
S7	27	19.47	39	0/0	Previous cerebral hemorrhage
S8	32	17.93	39	1/0	Severe myopia
S9	25	28.16	40	2/1	Previous cesarean sections
S10	44	17.72	40	0/2	Tocophobia
S11	39	24.02	39	3/2	Hip dysplasia
S12	34	21.19	39	0/0	Previous laparoscopic myomectomy
S13	35	24.28	39	1/1	Placenta previa
S14	26	24.24	39	1/0	Previous cesarean sections
S15	20	17.99	40	0/0	Breech presentation
S16	37	24.61	39	1/1	Previous cesarean section
S17	34	19.49	40	0/2	Fetal macrosomia
S18	32	29.30	42	0/0	Fetal macrosomia
S19	25	17.78	39	0/0	Fetal malformation (neck hemangioma)

**Table 2:** Percentages of bacterial genera identified in the uterine microbiota represented in more than 50% of the investigated subjects.

Genus	Abundance % (*)	Representation % (**)
*Cutibacterium*	9.35	100
*Pelomonas*	8.70	100
*Escherichia*	5.27	84
*Staphylococcus*	3.41	89
*Acinetobacter*	2.82	84
*Mesorhizobium*	2.07	95
*Bradyrhizobium*	1.96	95
*Streptococcus*	1.82	89
*Schlegelella*	1.60	89
*Dyella*	1.46	95
*Corynebacterium*	1.34	53

(*) The value is calculated as an average over all the samples. (**) Percentage of subjects where the genus abundance is ≥1.0%.

## Supplementary Materials

The following are available online at https://www.mdpi.com/2073-4425/10/12/971/s1, Figure S1: ASV rarefaction curves of bacterial 16S rRNA gene amplicon sequences, Figure S2: Faith index values related to the bacterial 16S rRNA gene amplicon sequences for the endometrial samples, Figure S3: NMDS plots based on UniFrac and Bray-Curtis matrixes, Table S1: Total number of sequences represented in ASVs, Table S2: Relative genera abundances identified for each patient’s endometrium.
