# Supplementary material for: Human Endometrial Microbiota at Term of Normal Pregnancies
Source: Genes (Basel). 2019 Nov 26;10(12):971. doi: 10.3390/genes10120971 (PMC6947671; doi:10.3390/genes10120971)
Supplement: Supplementary file 1 [file genes-10-00971-s001.pdf]

Supplementary Material

Human endometrial microbiota at term of normal pregnancies

Leoni C.[1,*], Ceci O.[2,*], Manzari C.[3*], Fosso B.[3], Volpicella M.[1,3], Ferrari A.[2], Fiorella P.[2], Pesole G.[1,3], Cicinelli E. [2,#], Ceci L.R.[3,#]

*) Equal contributors

[#]) Corresponding author

[1]Department of Biosciences, Biotechnologies and Biopharmaceutics, University of Bari "A. Moro", Via Orabona 4, 70126 Bari (Italy).

[2]2nd Unit of Obstetrics and Gynecology, Department of Biomedical Science and Human Oncology, University of Bari "A. Moro", Piazza G. Cesare, 70124 Bari (Italy).

[3]Institute of Biomembranes, Bioenergetics and Molecular Biotechnologies, CNR, Via Amendola 122/O, 70126 Bari (Italy).

Material Supplied

Table S1. Total number of sequences represented in ASVs.

Table S2. Relative genera abundances identified for each patient's endometrium.

Figure S1. ASV rarefaction curves of bacterial 16S rRNA gene amplicon sequences.

Figure S2. Faith index values related to the bacterial 16S rRNA gene amplicon sequences for the endometrial samples.

Figure S3. NMDS plots based on UniFrac and Bray-Curtis matrixes.

Table S1

Sample	N° of sequences	Sample	N° of sequences	Sample	N° of sequences
S5.1	15,283	S10.2	19,510	S18.3	30,629
S5.2	15,397	S14.1	19,716	S15.2	30,837
S14.2	15,397	S6.2	19,747	S8.1	31,317
S16.3	15,406	S7.3	20,394	S9.3	31,405
S19.2	15,833	S15.3	20,456	S13.1	31,719
S13.3	15,999	S13.2	20,636	S8.3	32,353
S2.3	16,403	S1.1	20,670	S11.1	32,927
S1.3	16,414	S4.1	21,214	S19.3	33,253
S16.1	17,422	S18.2	21,547	S7.2	35,325
S12.1	18,140	S17.1	21,604	S3.3	36,547
S17.3	18,303	S10.3	22,672	S8.2	38,336
S6.3	18,416	S17.2	23,126	S9.2	39,807
S4.3	18,580	S10.1	23,170	S12.2	40,932
S14.3	18,663	S15.1	24,396	S3.1	45,823
S7.1	18,866	S6.1	25,522	S18.1	54,583
S11.2	19,027	S5.3	25,628	S9.1	142,743
S3.2	19,114	S12.3	27,277		
S16.2	19,478	S11.3	29,754		

Table S1: Total number of sequences represented in ASV per each sample after the contaminants removal. S1-S19 indicate the different subjects. Samples are ordered according to the number of observed sequences.

Table S2

Genus / Subjects	S1	S2	S3	S4	S5	S6	S7	S8	S9	S10	S11	S12	S13	S14	S15	S16	S17	S18	S19	Average
Acidocella	0.69	1.79	0.72	0.97	0.26	0.73	1.01	0.84	0.44	0.75	0.71	0.73	0.96	1.13	0.75	0.83	0.94	0.50	0.41	0.80
Acinetobacter	0.65	1.83	6.54	2.12	1.23	3.90	1.57	2.09	3.00	1.38	1.08	1.06	1.11	1.13	16.72	5.03	0.78	0.89	1.48	2.82
Anaerococcus	0.21	0.14	0.04	0.44	1.31	1.16	0.27	0.31	0.29	0.04	0.55	1.06	0.24	0.08	0.35	0.09	0.13	0.93	0.18	0.41
Aquitalea	1.05	1.05	0.63	0.47	0.86	1.16	0.87	0.81	0.40	1.26	0.79	0.44	0.28	1.53	0.92	0.99	0.74	0.30	0.72	0.80
Bradyrhizobium	0.95	1.69	1.51	2.58	1.66	1.78	2.38	1.87	1.08	1.93	2.32	2.52	1.64	2.59	1.81	2.80	2.68	1.50	1.92	1.96
Chitinophaga	0.30	0.81	1.13	0.54	0.60	1.05	1.27	0.77	0.36	1.26	0.84	0.73	1.13	1.40	0.96	1.42	1.16	0.85	0.80	0.92
Comamonas	0.51	0.13	4.48	0.80	1.52	1.87	0.10	0.09	5.01	0.19	0.15	0.24	0.54	1.07	4.78	0.44	0.14	0.58	0.12	1.20
Corynebacterium	1.92	0.43	0.90	1.71	2.86	2.29	1.78	1.18	0.36	0.76	1.60	0.56	1.53	0.54	0.72	1.33	0.99	3.61	0.34	1.34
Dechloromonas	0.47	0.07	0.01	0.32	2.29	1.09	0.25	0.04	3.40	0.42	0.14	0.85	0.68	1.40	2.57	0.68	0.04	0.89	0.00	0.82
Diaphorobacter	0.43	0.44	0.06	1.75	0.18	2.29	0.13	0.03	3.31	0.57	0.01	0.06	0.27	0.43	2.96	0.74	0.23	0.21	0.22	0.75
Dyella	2.76	1.28	1.25	1.67	0.60	1.98	1.70	1.28	0.65	1.48	1.38	1.33	1.56	1.60	1.22	1.82	1.85	1.17	1.16	1.46
Escherichia	11.65	3.55	0.72	1.16	13.71	5.95	0.94	10.64	0.52	5.80	6.78	11.06	5.07	4.92	3.04	1.98	8.15	1.80	2.59	5.27
Geothrix	0.68	0.79	0.76	0.68	0.64	1.05	1.51	0.76	0.43	0.88	1.00	0.82	0.47	1.10	1.07	1.20	0.92	0.79	0.75	0.86
Lactobacillus	0.00	0.00	0.03	0.19	0.42	0.10	1.40	0.01	0.14	0.13	0.05	15.22	6.26	0.00	0.08	0.14	0.19	0.32	16.18	2.15
Mesorhizobium	1.95	2.59	2.57	2.56	1.26	2.40	1.92	2.12	1.04	2.24	1.98	2.26	2.10	2.48	1.66	2.49	2.64	0.98	2.01	2.07
Methylobacterium	0.52	0.68	0.77	1.03	0.62	0.45	0.33	0.51	0.06	0.22	0.21	0.39	0.82	0.28	0.46	0.65	0.23	2.98	0.20	0.60
Novosphingobium	0.48	0.15	0.14	0.32	5.88	0.44	0.45	0.01	0.30	0.21	0.46	1.25	1.18	3.37	0.07	0.05	0.00	1,45	0.00	0.85
other	10.53	13.07	8.16	11.39	8.12	9.20	9.09	8.61	5.14	9.75	7.86	9.03	10.75	10.06	9.25	8.94	9.42	12.33	7.24	9.37
Pelomonas	9.96	10.56	8.27	12.98	7.50	9.80	9.97	6.60	4.78	9.38	7.47	6.96	9.86	9.31	7.71	11.24	8.93	6.58	7.45	8.70
Propionibacterium	13.40	5.81	4.26	10.25	11.87	6.15	17.13	10.02	2.12	11.31	20.98	9.62	10.86	5.33	3.79	5.50	10.63	10.70	7.96	9.35
Pseudomonas	0.97	1.36	0.70	1.17	0.36	1.45	0.72	0.76	35.58	0.44	0.50	0.39	0.64	0.33	1.36	0.29	0.58	0.99	0.20	2.57
Rhodococcus	0.76	0.43	0.15	0.31	0.64	0.53	1.04	0.25	0.41	1.09	0.72	0.39	0.42	0.39	0.30	0.31	0.59	0.37	0.08	0.48
Schlegelella	2.14	1.62	1.07	2.45	1.40	1.27	1.48	0.99	1.47	1.71	1.62	1.29	2.09	1.82	1.77	2.26	1.72	0.76	1.45	1.60
Sphingobium	0.00	0.67	11.11	0.06	0.00	0.06	0.04	0.01	7.87	0.15	0.01	0.02	0.00	0.00	1.03	0.15	0.07	0.08	0.00	1.12
Sphingomonas	1.27	0.10	1.19	0.48	0.58	0.25	0.55	1.20	0.28	0.58	0.23	0.50	0.46	0.49	0.85	0.21	0.52	0.60	0.20	0.56
Staphylococcus	2.27	2.27	1.53	6.47	3.46	4.99	2.99	3.40	0.88	2.37	1.66	1.64	3.92	1.10	0.78	3.26	2.07	2.17	17.62	3.41
Streptococcus	5.03	2.59	2.32	1.10	2.68	1.45	1.90	2.72	0.29	1.66	1.66	2.53	1.61	0.64	1.75	1.16	1.42	1.25	0.81	1.82
unknown_Bacteria	13.76	24.32	23.22	17.67	14.32	21.38	22.81	16.60	8.53	25.37	21.81	14.99	19.20	29.82	20.78	28.49	27.52	15.85	17.65	20.22
unknown_Deltaproteobacteria	1.92	2.41	1.15	0.67	1.59	2.17	1.72	1.27	0.76	1.74	1.83	1.00	1.52	2.39	0.97	1.70	1.13	1.66	1.68	1.54
unknown_Acidobacteriales	1.24	2.42	1.78	1.08	1.57	2.08	2.20	1.84	0.81	2.75	1.96	1.23	1.60	2.38	1.11	1.69	1.71	1.40	1.55	1.71
unknown_Rhizobiales	0.23	0.92	1.13	0.69	0.53	0.82	0.84	0.86	0.36	0.78	1.43	0.46	1.00	0.89	0.59	0.53	1.26	1.07	0.72	0.80

Table S2. Relative genera abundances identified for each patient's endometrium (only genera with abundances higher than 1% are reported). S1-S19 indicate the different subjects.

Figure S1

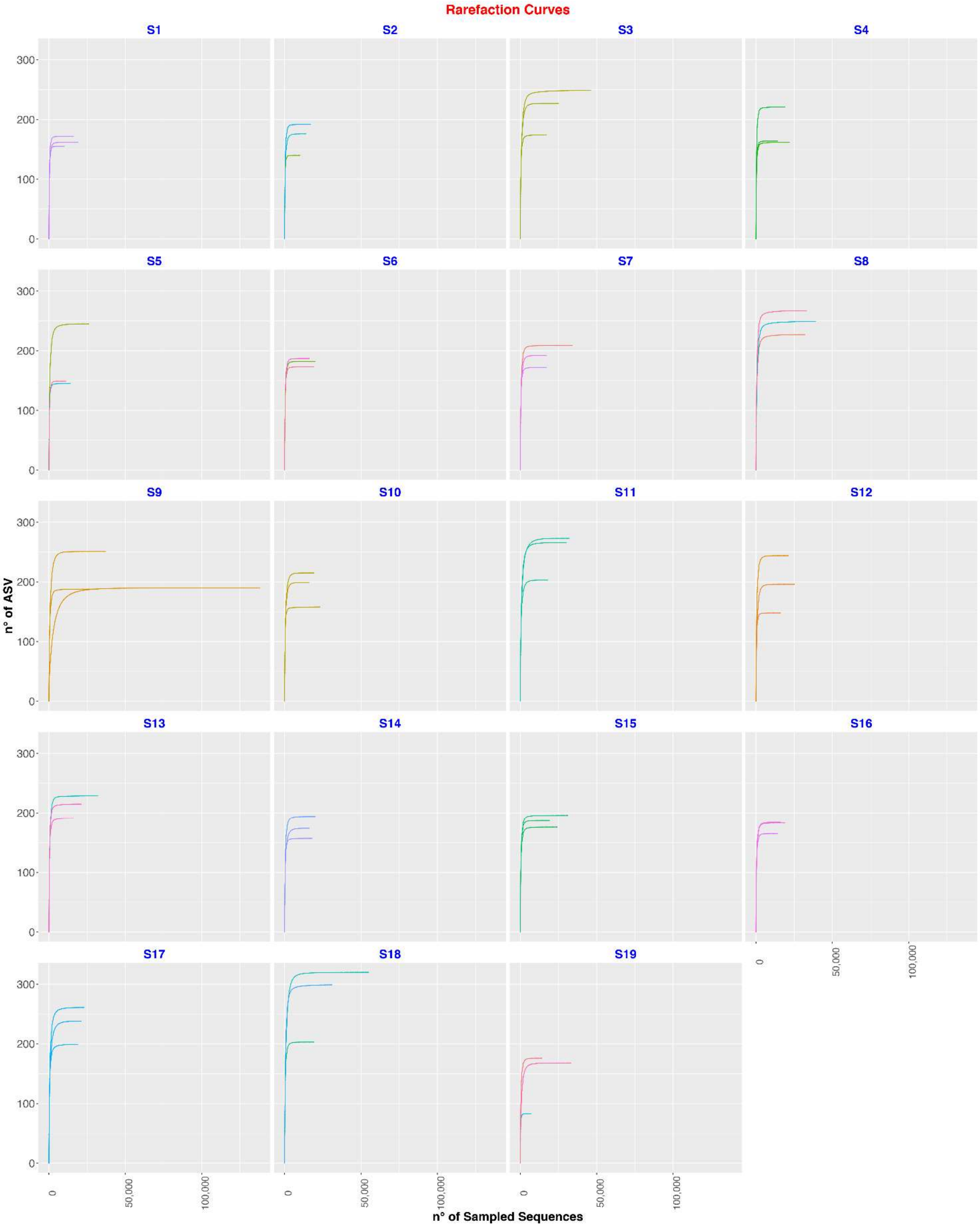

Figure S1. ASV rarefaction curves of bacterial 16S rRNA gene amplicon sequences for endometrial samples. S1-S19 indicate the different subjects.

Figure S2

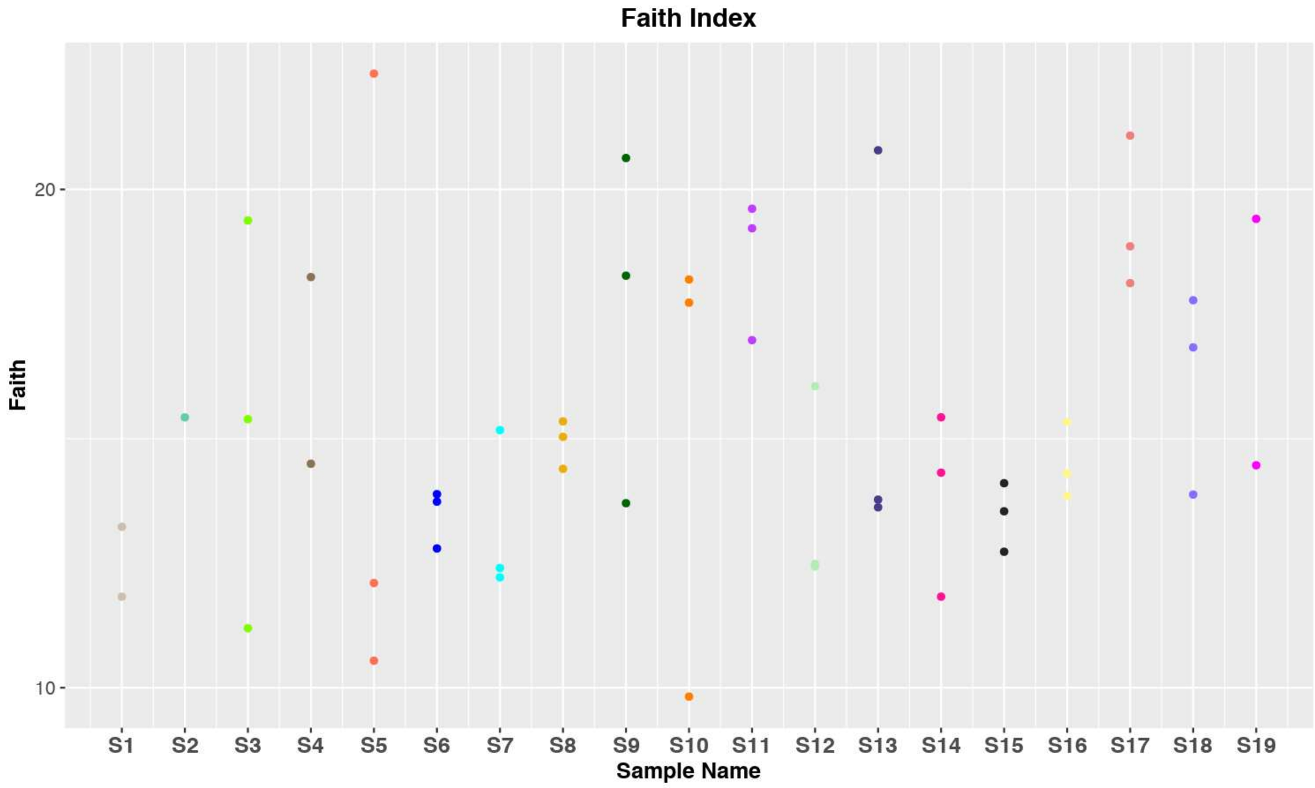

Figure S2. Faith index values related to the bacterial 16S rRNA gene amplicon sequences for the endometrial samples. S1-S19 indicate the different subjects. Coloured dots correspond to single V5-V6 amplicon sequencing sample.

Figure S3

A

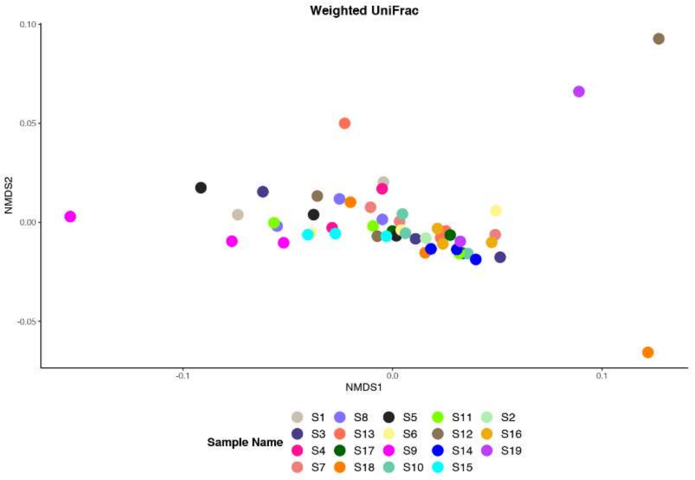

B

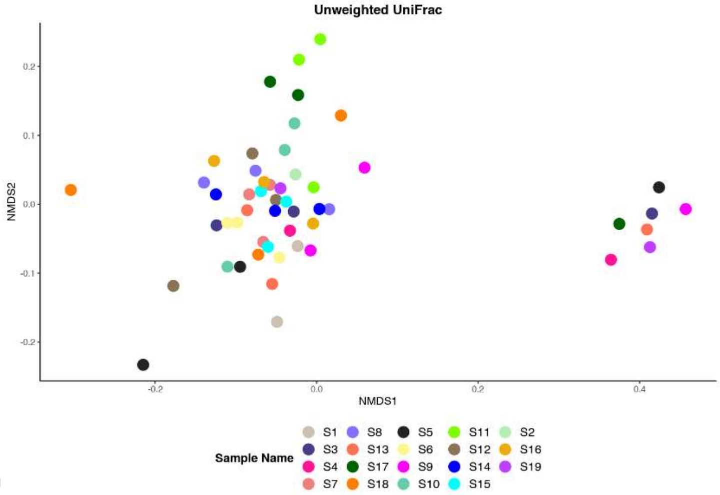

C

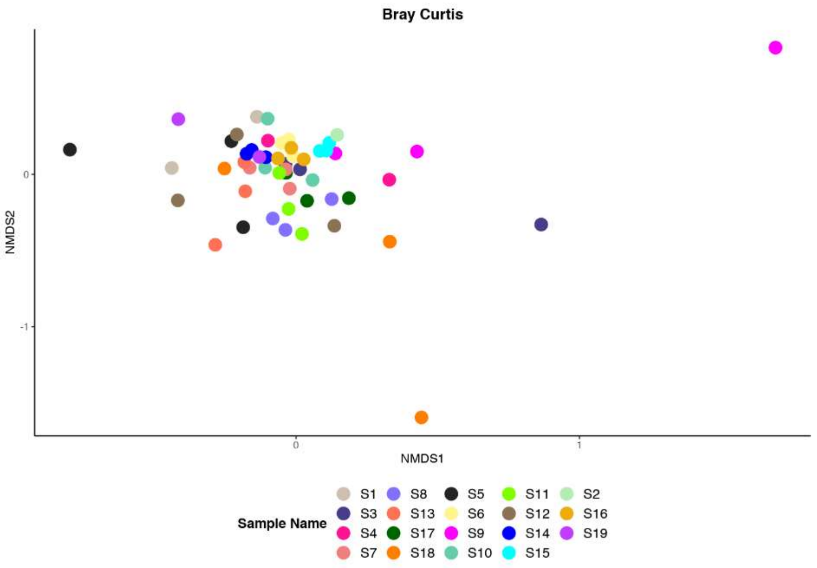

Figure S3. NMDS plots based on UniFrac weighted dissimilarity matrix (A), UniFrac unweighted dissimilarity matrix (B) and Bray-Curtis matrix (C). S1-S19 indicate the different subjects. Coloured dots correspond to single V5-V6 amplicon sequencing sample.
